# Disentanglement Dynamics in Nonequilibrium Environments

**DOI:** 10.3390/e24101330

**Published:** 2022-09-21

**Authors:** Mingli Chen, Haonan Chen, Tao Han, Xiangji Cai

**Affiliations:** School of Science, Shandong Jianzhu University, Jinan 250101, China

**Keywords:** open quantum system, decoherence, disentanglement

## Abstract

We theoretically study the non-Markovian disentanglement dynamics of a two-qubit system coupled to nonequilibrium environments with nonstationary and non-Markovian random telegraph noise statistical properties. The reduced density matrix of the two-qubit system can be expressed as the Kraus representation in terms of the tensor products of the single qubit Kraus operators. We derive the relation between the entanglement and nonlocality of the two-qubit system which are both closely associated with the decoherence function. We identify the threshold values of the decoherence function to ensure the existences of the concurrence and nonlocal quantum correlations for an arbitrary evolution time when the two-qubit system is initially prepared in the composite Bell states and the Werner states, respectively. It is shown that the environmental nonequilibrium feature can suppress the disentanglement dynamics and reduce the entanglement revivals in non-Markovian dynamics regime. In addition, the environmental nonequilibrium feature can enhance the nonlocality of the two-qubit system. Moreover, the entanglement sudden death and rebirth phenomena and the transition between quantum and classical nonlocalities closely depend on the parameters of the initial states and the environmental parameters in nonequilibrium environments.

## 1. Introduction

Coherence and entanglement are two basic quantum features of nonclassical systems, which play vital roles in quantum mechanical community as specific resources ranging from fundamental questions to wide applications in quantum computing, quantum metrology and quantum information science [1,2,3,4,5,6]. It is known that any quantum system loses quantum features during time evolution resulting from the unavoidable couplings between the system and the environments. The loss of quantum features induced by the environments is considered as a fundamental obstacle to the construction of quantum information processors and the realization of ultrafast quantum computation. The study of decoherence and disentanglement dynamics of open quantum systems can help us further expand the understanding of the environmental effects on the dynamical evolution of the quantum systems and the real origins of the loss of quantum features and quantum-classical transition, which has potential applications in preserving quantum features against the environmental noise and in realizing quantum manipulation and control and quantum measurement [7,8,9,10,11,12,13,14,15,16,17,18,19,20,21,22].

During the last few decades, the dynamics of open quantum systems is usually investigated within Markov approximation, i.e., when we neglect the memory effect of the dynamical evolution and the higher-order environmental correlations, described by a formally solvable Lindblad type master equation. With the development of the experimental technique, it has been observed accurately that the dynamical evolution of open quantum systems is closely associated with a flow of information from the environments back into the system. For instance, the electronic energy transfer processes in photosynthesis and the dynamical decoherence in quantum bit systems exhibit strong non-Markovian behavior [23,24,25,26,27,28]. In recent decades, increasing attention has been attracted to theoretically studying the dynamics of open quantum systems beyond the framework of Markovian approximation [29,30,31,32,33,34,35,36,37,38], and there have been well established theoretical approaches to study the non-Markovian dynamics of open quantum systems within the framework of classical and quantum treatments [39,40,41,42,43,44,45,46,47,48,49,50,51,52,53,54,55,56,57,58,59,60,61,62,63]. Meanwhile, the coherence and entanglement revivals and entanglement sudden death and rebirth phenomena have been extensively studied theoretically and observed experimentally in the presence of the non-Markovian behavior in the quantum dynamics [64,65,66,67,68,69,70].

Recently, the nonequilibrium feature of the environments in many crucial dynamical processes has been experimentally observed. In these processes, the environmental initial states caused by the interaction with the quantum systems cannot become stationary in time, which corresponds to the environments around the quantum systems being out of equilibrium [71,72,73,74]. Random telegraph noise (RTN) is an important classical non-Gaussian noise, which has theoretically simulated the environmental influences on open quantum systems, such as single molecule fluorescence [75,76], disentanglement, decoherence and frequency modulation processes in the presence of low-frequency $1/f^{\alpha }$ noise [77,78,79,80,81,82,83]. Furthermore, the quantum dynamics that are stochastically driven by the classical fluctuating field displaying random telegraph fluctuations have been investigated experimentally [84,85]. The previous investigations usually assumed that the RTN displays stationary and Markovian properties. As a matter of fact, the stationary and Markovian assumption is only an idealization of both real internal fluctuations and external disturbances, and the real properties of the fluctuations and disturbances induced by the environments are neither stationary nor Markovian. Based on this fact, the stationary non-Markovian RTN and the nonstationary non-Markovian RTN with an exponential memory kernel have been successively put forward and discussed [86,87], and the latter has been widely used to study the relevant issues on the dynamics of open quantum systems in nonequilibrium environments [87,88,89,90,91,92,93]. Studying the environmental nonequilibrium effects on quantum coherence due to the significant role in the dynamical evolution of the open quantum systems has increasingly drawn much attention, and the theoretical results demonstrate that nonequilibrium environments cause the energy levels shift of the quantum system and delay the transition critical time of decoherence from classical to quantum [87,88,92,93]. To the best of our knowledge, the disentanglement dynamics in nonequilibrium environments has not been studied yet. Meanwhile, some other important physical questions arise naturally and should be further addressed: Can we find the close relations between the local decoherence and nonlocal entanglement and quantum nonlocality of open quantum systems in nonequilibrium environments? How do the environmental nonequilibrium feature influence the disentanglement dynamics and quantum nonlocality of open quantum systems? Are there the entanglement sudden death and rebirth phenomena or the transition between quantum and classical nonlocalities in nonequilibrium environments?

In this paper, we theoretically study the non-Markovian dynamics of a two-qubit system interacting with nonequilibrium environments, which display nonstationary and non-Markovian RTN statistical properties. The two-qubit system consists of two noncoupling identical single qubits independently interacting with its local nonequilibrium environment, of which the reduced density matrix can be expressed as the Kraus representation in terms of the tensor products of the single qubit Kraus operators. We derive the relations between the decoherence function and the entanglement quantified by the concurrence and the nonlocality characterized by the Bell function. We identify the threshold values of the decoherence function to ensure the existences of the concurrence and nonlocal quantum correlations at an arbitrary evolution time for the two-qubit system prepared initially in the composite Bell states and the Werner states, respectively. It is demonstrated that the environmental nonequilibrium feature can suppress both the decoherence and disentanglement dynamics and that it can reduce the coherence and entanglement revivals in non-Markovian dynamics regime. In addition, the environmental nonequilibrium feature can enhance the nonlocality of the two-qubit system. Moreover, the phenomena of entanglement sudden death and rebirth and the transition between quantum and classical nonlocalities are closely dependent on the parameters of the initial states in nonequilibrium environments.

This paper is organized as follows. In Section 2, we introduce the theoretical framework of non-Markovian disentanglement dynamics in nonequilibrium environments. We employ the non-Markovianity, concurrence and Bell function to describe the non-Markovian two-qubit disentanglement dynamics in nonequilibrium environments. In Section 3, we discuss the numerical results of the non-Markovian two noninteracting qubit disentanglement dynamics in nonequilibrium environments with nonstationary and non-Markovian RTN statistical properties. In Section 4, we present the conclusions from the present study.

## 2. Theoretical Framework

### 2.1. Non-Markovian Disentanglement Dynamics of a Two-Qubit System

We consider a two-qubit system *T* consisting of two noninteracting identical single qubits *A* and *B* independently interacting with its nonequilibrium environment exhibiting nonstationary and non-Markovian RTN statistical properties, respectively. The single qubit *S*(S=A,B) can be characterized as a two-level system with the states |1〉 and |0〉. The environmental effects lead to the stochastic fluctuations in the Hamiltonian of the two-qubit system as

(1) $$H_{T}\left(t\right)=H_{S}\left(t\right)⊗I+I⊗H_{S}\left(t\right),$$

where *I* denotes the identity matrix and $H_{S}\left(t\right)$ is the stochastic Hamiltonian of the single qubit system *S* coupled to its local nonequilibrium environment *E*, written as

(2) $$H_{S}\left(t\right)=\frac{\hbar }{2}\left[\omega_{0}+\xi \left(t\right)\right]\sigma_{z},$$

with $\omega_{0}$ denoting the frequency difference of the single qubit system, $\sigma_{z}=\left|1\rangle \langle 1\right|-\left|0\rangle \langle 0\right|$ being the Pauli matrix in the single qubit basis $B_{S}=\{|1\rangle ,|0\rangle \}$ and the environmental noise ξ(t) subject to a generalized RTN stochastic process.

Due to the fact that the two single qubits of the system do not interact with each other initially, the dynamics of the two-qubit system can be obtained from that of a singe qubit system by means of the Kraus representation [40,94]. Thus, to derive the dynamics of the two-qubit system, we first consider that of the single qubit system. Because the environmental effects lead to the stochastic fluctuations in the frequency difference between the states |1〉 and |0〉, the single qubit system undergoes pure decoherence during its dynamical evolution. By taking an average over the environmental noise ξ(t), we can express the reduced density matrix of the single qubit system in the Kraus representation as

(3) $$\begin{array}{c} \rho_{S}\left(t\right)=\sum_{\mu =1}^{2}K_{S\mu }\left(t\right)\rho_{S}\left(0\right)K_{S\mu }^{†}\left(t\right), \end{array}$$

with the single qubit Kraus operators $K_{S\mu }$

(4) $$K_{S1}\left(t\right)=\left(\begin{array}{cc} 1 & 0\\ 0 & e^{i\omega_{0}t}F\left(t\right) \end{array}\right),K_{S2}\left(t\right)=\left(\begin{array}{cc} 0 & 0\\ 0 & \sqrt{1-{\left|F\left(t\right)\right|}^{2}} \end{array}\right),$$

where $F\left(t\right)=\langle \exp\left[i\int_{0}^{t}dt^{\prime }\xi \left(t^{\prime }\right)\right]\rangle$ denotes the decoherence function with 〈⋯〉 being the average taken over the environmental noise ξ(t). The diagonal elements of the reduced density matrix of the single qubit system are time independent and the off diagonal elements evolve with time

(5) $$\begin{array}{cc} \rho_{00}\left(t\right) & =\rho_{00}\left(0\right),\\ \rho_{11}\left(t\right) & =1-\rho_{00}\left(t\right),\\ \rho_{01}\left(t\right) & =\rho_{10}^{*}\left(t\right)=\rho_{01}\left(0\right)e^{i\omega_{0}t}F\left(t\right). \end{array}$$

Because of the nonstationary statistical property of the environmental noise, the decoherence function is complex. The dynamical evolution of the single qubit system is closely associated with the decoherence rate γ(t)=−Re[(d/dt)F(t)/F(t)] and the frequency shift s(t)=−Im[(d/dt)F(t)/F(t)] [87,88].

In the presence of the standard RTN, the amplitude of the environmental noise jumps randomly with the switching rate λ between the values ±ν. The ratio ν/λ describes the environmental coupling and there are two important dynamic regimes identified: the weak coupling regime ν/λ<1 and the strong coupling regime ν/λ>1. The statistical properties of the standard RTN is time-homogeneous, Markovian and stationary. Physically, the statistical properties of the generalized RTN can be extracted from that of the standard RTN based on classical probability theory [95]. In the following, we introduce a class of time-homogeneous, non-Markovian and nonstationary RTN (see Appendix A).

For the time-homogeneous generalized RTN process, the environmental non-Markovian property is described by a generalized master equation for the time evolution of the conditional probability [86]

(6) $$\frac{\partial }{\partial t}P\left(\xi ,t|\xi^{\prime },t^{\prime }\right)=\int_{t^{\prime }}^{t}K\left(t-\tau \right)\lambda TP\left(\xi ,\tau |\xi^{\prime },t^{\prime }\right)d\tau ,$$

where K(t−τ) is the memory kernel of the environmental noise, and the conditional probability $P(\xi ,t|\xi^{\prime },t^{\prime })$ and transition matrix T are respectively expressed as

(7) $$P\left(\xi ,t|\xi^{\prime },t^{\prime }\right)=\left(\begin{array}{c} P(+\nu ,t|\xi^{\prime },t^{\prime })\\ P(-\nu ,t|\xi^{\prime },t^{\prime }) \end{array}\right),T=\left(\begin{array}{cc} -1 & 1\\ 1 & -1 \end{array}\right).$$

Physically, the extraction of a subensemble non-Markovian processes with the memory effect taken into account means that the statistical properties of the environmental noise depend on previous history. When the environmental noise is memoryless, i.e., K(t−τ)=δ(t−τ), the non-Markovian RTN recovers the Markovian one and its memory effect vanishes. By means of the Laplace transformation $\tilde{P}\left(\xi ,s|\xi^{\prime },t^{\prime }\right)=\int_{0}^{\infty }P\left(\xi ,t|\xi^{\prime },t^{\prime }\right)e^{-st}dt$, the conditional probability in Equation (6) can be analytically expressed as

(8) $$P\left(\xi ,t|\xi^{\prime },t^{\prime }\right)=\left[I+\frac{1-P(t-t^{\prime })}{2}T\right]P\left(\xi ,t^{\prime }|\xi^{\prime },t^{\prime }\right),$$

where the auxiliary probability function $P\left(t-t^{\prime }\right)=L^{-1}\left[e^{-st^{\prime }}\tilde{P}\left(s\right)\right]$ with $\tilde{P}\left(s\right)=1/\left[s+2\lambda \tilde{K}\left(s\right)\right]$ and $L^{-1}$ denotes the inverse Laplace transform. Due to the fact that the memory kernel in the conditional probability depends on the time difference, the environmental noise is subject to an homogeneous stochastic process.

The environmental nonstationary property arises from the initial distribution

(9) $$P\left(\xi_{0},0\right)=\frac{1}{2}\left(1+a\right)\delta_{\xi_{0},\nu }+\frac{1}{2}\left(1-a\right)\delta_{\xi_{0},-\nu },$$

where *a* is the nonstationary parameter and −1≤a≤1. Correspondingly, the nonstationary one-point probability distribution satisfies

(10) $$\begin{array}{cc} P(\xi ,t) & =\sum_{\xi_{0}}P\left(\xi ,t|\xi_{0},0\right)P\left(\xi_{0},0\right)=\frac{1}{2}\left[1+aP\left(t\right)\right]\delta_{\xi ,\nu }+\frac{1}{2}\left[1-aP\left(t\right)\right]\delta_{\xi ,-\nu }. \end{array}$$

Physically, the extraction of a subensemble nonstatioanry processes with initial nonstationary distribution means that the statistical property of the environmental noise is time dependent initially, which corresponds to the environment being in a certain initial nonequilibrum state [95]. For the case a=0, the environmental noise only displays stationary property corresponding to that the environment is in equilibrium [87,88].

According to the non-Markovian and nonstationary properties described above, the statistical characteristics of the environmental noise ξ(t) are described by the first and second-order moments

(11) $$\begin{array}{cc} \langle \xi (t)\rangle & =a\nu P(t),\\ \langle \xi \left(t\right)\xi \left(t^{\prime }\right)\rangle & =\nu^{2}P\left(t-t^{\prime }\right). \end{array}$$

where $L^{-1}$ denotes the inverse Laplace transform. According to the Bayes’ theorem, the environmental higher odd- and even-order moments satisfy the factorization

(12) $$\begin{array}{cc} \langle \xi \left(t_{1}\right)\xi \left(t_{2}\right)\cdots \xi \left(t_{2n-1}\right)\rangle & =\langle \xi \left(t_{1}\right)\xi \left(t_{2}\right)\rangle \langle \xi \left(t_{3}\right)\xi \left(t_{4}\right)\rangle \cdots \langle \xi \left(t_{2n-1}\right)\rangle \\ & =av^{2n-1}P\left(t_{1}-t_{2}\right)\cdots P\left(t_{2n-1}\right),\\ \langle \xi \left(t_{1}\right)\xi \left(t_{2}\right)\cdots \xi \left(t_{2n}\right)\rangle & =\langle \xi \left(t_{1}\right)\xi \left(t_{2}\right)\rangle \langle \xi \left(t_{3}\right)\xi \left(t_{4}\right)\rangle \cdots \langle \xi \left(t_{2n-1}\right)\xi \left(t_{2n}\right)\rangle \\ & =v^{2n}P\left(t_{1}-t_{2}\right)\cdots P\left(t_{2n-1}-t_{2n}\right), \end{array}$$

for the order of the time instants $t_{1}>\cdots >t_{2n}$(n≥2). This factorization relation for the higher-order correlation functions recovers to the case that the RTN process exhibits only stationary property due to the vanishing of the odd moments of the environmental noise [86,96]. It is worth mentioning that nonstationary property of the environmental noise only influences the odd-order moments due to our extraction of the subensemble time-homogeneous nonstatioanry processes made above. If the environmental noise ξ(t) exhibits stationary statistical property, namely, a=0, the odd-order moments in its statistical characteristics will vanish [86,96].

We consider the case that the environmental memory kernel is of an exponential form as $K\left(t-\tau \right)=\kappa e^{-\kappa (t-\tau )}$ with κ denoting the memory decay rate. The smaller is the decay rate κ, the stronger is the environmental non-Markovian property. For the case κ→+∞, namely, the memoryless case K(t−τ)=δ(t−τ), the environmental noise only exhibits Markovian property. Based on the exponential form of the memory kernel, each order moment of the environmental noise obeys the closed second-order differential relation

(13) $$\frac{d^{2}}{dt^{2}}\langle \xi \left(t\right)\cdot \cdot \cdot \xi \left(t_{n}\right)\rangle +\kappa \frac{d}{dt}\langle \xi \left(t\right)\cdot \cdot \cdot \xi \left(t_{n}\right)\rangle +2\kappa \lambda \langle \xi \left(t\right)\cdot \cdot \cdot \xi \left(t_{n}\right)\rangle =0.$$

In terms of Equation (13) and the generalized Dyson expansion for the decoherence function

(14) $$F\left(t\right)=1+\sum_{n=1}^{\infty }i^{n}\int_{0}^{t}dt_{1}\cdot \cdot \cdot \int_{0}^{t_{n-1}}dt_{n}\langle \xi \left(t_{1}\right)\cdot \cdot \cdot \xi \left(t_{n}\right)\rangle ,$$

for all the time instants $t>t_{1}>\cdot \cdot \cdot >t_{n}>0$, we obtain a closed third-order differential equation for the decoherence function in the single qubit system

(15) $$\frac{d^{3}}{dt^{3}}F\left(t\right)+\kappa \frac{d^{2}}{dt^{2}}F\left(t\right)+\left(2\kappa \lambda +\nu^{2}\right)\frac{d}{dt}F\left(t\right)+\kappa \nu^{2}F\left(t\right)=0,$$

with the initial conditions F(0)=1, (d/dt)F(0)=iaν and $\left(d^{2}/dt^{2}\right)F\left(0\right)=-\nu^{2}$. Correspondingly, the decoherence function for the single qubit system can be exactly expressed as [88]

(16) $$\begin{array}{c} F\left(t\right)=L^{-1}\left[F\left(s\right)\right],\;F\left(s\right)=\frac{s^{2}+\kappa s+2\kappa \lambda +ia\nu \left(s+\kappa \right)}{s^{3}+\kappa s^{2}+\left(2\kappa \lambda +\nu^{2}\right)s+\kappa \nu^{2}}. \end{array}$$

We now construct the reduced density matrix of the two-qubit system in the standard product basis $B_{T}=\{|1\rangle =|11\rangle ,|2\rangle =|10\rangle ,|3\rangle =|01\rangle ,|4\rangle =\left|00\rangle \right\}$. Based on the two-qubit basis and by taking an average over the environmental noise, we express the reduced density matrix of the two-qubit system in the Kraus representation as

(17) $$\begin{array}{c} \rho_{T}\left(t\right)=\sum_{\mu =1}^{4}K_{T\mu }\left(t\right)\rho_{T}\left(0\right)K_{T\mu }^{†}\left(t\right), \end{array}$$

where the two-qubit Kraus operators $K_{T\mu }\left(t\right)=K_{S\nu }\left(t\right)⊗K_{S\upsilon }\left(t\right)\left(\nu ,\upsilon =1,2\right)$ are the tensor products of the single qubit Kraus operators

(18) $$\begin{array}{cc} K_{T1}\left(t\right) & =\left(\begin{array}{cc} 1 & 0\\ 0 & e^{i\omega_{0}t}F\left(t\right) \end{array}\right)⊗\left(\begin{array}{cc} 1 & 0\\ 0 & e^{i\omega_{0}t}F\left(t\right) \end{array}\right),\\ K_{T2}\left(t\right) & =\left(\begin{array}{cc} 1 & 0\\ 0 & e^{i\omega_{0}t}F\left(t\right) \end{array}\right)⊗\left(\begin{array}{cc} 1 & 0\\ 0 & \sqrt{1-{\left|F\left(t\right)\right|}^{2}} \end{array}\right),\\ K_{T3}\left(t\right) & =\left(\begin{array}{cc} 0 & 0\\ 0 & \sqrt{1-{\left|F\left(t\right)\right|}^{2}} \end{array}\right)⊗\left(\begin{array}{cc} 1 & 0\\ 0 & e^{i\omega_{0}t}F\left(t\right) \end{array}\right),\\ K_{T4}\left(t\right) & =\left(\begin{array}{cc} 0 & 0\\ 0 & \sqrt{1-{\left|F\left(t\right)\right|}^{2}} \end{array}\right)⊗\left(\begin{array}{cc} 0 & 0\\ 0 & \sqrt{1-{\left|F\left(t\right)\right|}^{2}} \end{array}\right). \end{array}$$

Due to the pure decoherence, the diagonal elements of the reduced density matrix are time-independent and the off-diagonal elements decay with time monotonously (Markovian behavior) or non-monotonously (non-Markovian behavior). According to the two-qubit Kraus operators expression for the reduced density matrix in Equation (17), the diagonal elements do not evolve with time

(19) $$\begin{array}{cc} \rho_{11}\left(t\right) & =\rho_{11}\left(0\right),\\ \rho_{22}\left(t\right) & =\rho_{22}\left(0\right),\\ \rho_{33}\left(t\right) & =\rho_{33}\left(0\right),\\ \rho_{44}\left(t\right) & =1-[\rho_{11}\left(0\right)+\rho_{22}\left(0\right)+\rho_{33}\left(0\right)], \end{array}$$

and time-dependent off diagonal elements can be written as

(20) $$\begin{array}{cc} \rho_{21}\left(t\right) & =\rho_{12}^{*}\left(t\right)=\rho_{21}\left(0\right)e^{i\omega_{0}t}F\left(t\right),\\ \rho_{31}\left(t\right) & =\rho_{13}^{*}\left(t\right)=\rho_{31}\left(0\right)e^{i\omega_{0}t}F\left(t\right),\\ \rho_{32}\left(t\right) & =\rho_{23}^{*}\left(t\right)=\rho_{32}\left(0\right){\left|F\left(t\right)\right|}^{2},\\ \rho_{41}\left(t\right) & =\rho_{14}^{*}\left(t\right)=\rho_{41}\left(0\right)e^{i(2\omega_{0})t}F^{2}\left(t\right),\\ \rho_{42}\left(t\right) & =\rho_{24}^{*}\left(t\right)=\rho_{42}\left(0\right)e^{i\omega_{0}t}F\left(t\right),\\ \rho_{43}\left(t\right) & =\rho_{34}^{*}\left(t\right)=\rho_{43}\left(0\right)e^{i\omega_{0}t}F\left(t\right). \end{array}$$

By taking the optimization over all pairs of initial states, the non-Markovianity quantifying the flow of information exchange between the two-qubit system and environment can be expressed as [30,97]

(21) $$\begin{array}{cc} N_{T} & =\max_{\rho_{T}^{1,2}\left(0\right)}\int_{\frac{dD}{dt}>0}\frac{d}{dt}D\left(\rho_{T}^{1}\left(t\right),\rho_{T}^{2}\left(t\right)\right)dt=-2\int_{\gamma (t)<0}\gamma \left(t\right){\left|F\left(t\right)\right|}^{2}dt, \end{array}$$

where $D\left(\rho_{T}^{1},\rho_{T}^{2}\right)=\frac{1}{2}\mathrm{tr}\left|\rho_{T}^{1}-\rho_{T}^{2}\right|$ denotes the trace distance between the two-qubit states $\rho_{T}^{1}$ and $\rho_{T}^{2}$ and the optimal pair of initial states can be chosen as the maximally entangled states of super-decoherent Bell states $|\psi_{\pm }\left(0\right)\rangle =(|00\rangle \pm |11\rangle )/\sqrt{2}$ or sub-decoherent Bell states $|\varphi_{\pm }\left(0\right)\rangle =(|01\rangle \pm |10\rangle )/\sqrt{2}$ [98,99]. The two-qubit dynamics display non-Markovian behavior if the decoherence rate γ(t) takes negative values in some time intervals.

### 2.2. Relations between Local Decoherence and Nonlocal Entanglement and Quantum Nonlocality

Due to the environmental effects on its evolution, the two-qubit system undergoes dynamical disentanglement. Since the two single qubits of the system do not interact with each other, the dynamics of the two-qubit system can be obtained from that of a singe qubit system, as we derived above. Thus, the local decoherence described by the decoherence function F(t) plays an important role in the dynamics of the two-qubit system as that in a single qubit system [100,101]. Can we find the close relations between the local decoherence and nonlocal entanglement and quantum nonlocality of the two-qubit sytem in nonequilibrium environments? Are there the entanglement sudden death and rebirth phenomena or the transition between quantum and classical nonlocalities of the two-qubit system in nonequilibrium environments? To further study the effects of the local decoherence on the nonlocal entanglement and quantum nonlocality of the two-qubit sytem, we use the concurrence C(t) and the Clauser-Horne-Shimony-Holt (CHSH) form of Bell function B(t) to quantify the entanglement and quantum nonlocality of the two-qubit system (see Appendix B), respectively [94,102,103].

In the following, we derive the relations between the decoherence function and the entanglement quantified by the concurrence and the nonlocality characterized by the Bell function for the two-qubit system initially prepared in the composite Bell states and Werner states with an *X* structure density matrix, respectively [104,105]. In contrast to the previous investigations [82,100] that only discussed the threshold values of the initial state parameters for the existences of the concurrence and quantum nonlocality initially, we not only discuss the initial threshold values of the state parameters but also discuss the threshold values of the decoherence function for the existences of the concurrence and quantum nonlocality at an arbitrary time *t*.

We first focus on the initial states of the system in the composite Bell states of the form [106]

(22) $$\rho \left(0\right)=\frac{1+c}{2}|\psi_{\pm }\left(0\right)\rangle \langle \psi_{\pm }\left(0\right)|+\frac{1-c}{2}|\varphi_{\pm }\left(0\right)\rangle \langle \varphi_{\pm }\left(0\right)|,$$

where the initial state parameter *c* is real and satisfies −1≤c≤1. It has, by studying the quantum mutual information, quantum discord and classical correlations of the dynamics, which demonstrates that for the initial states in Equation (22) there is a sudden transition from classical to quantum decoherence for the two-qubit system coupled to a nonequilibrium environment exhibiting generalized RTN property, and the nonequilibrium feature of the environment can delay the critical time of the transition of decoherence from classical to quantum [92]. The concurrence at time *t* for the two-qubit system prepared in the initial states of Equation (22) can be reduced to

(23) $$C\left(t\right)=\max\left\{0,\frac{1+|c|}{2}{\left|F\left(t\right)\right|}^{2}-\frac{1-|c|}{2}\right\}.$$

The initial concurrence of the two-qubit system prepared in the composite Bell states in Equation (22) can be expressed as C(0)=|c|, since the initial value of the decoherence function satisfies F(0)=1. Therefore, the entanglement of the two-qubit system exists except for the special case c=0. For the case −1≤c<0, the concurrence at time *t* exists if the threshold value of the decoherence function satisfies $\left|F\left(t\right)|>\right|F_{\mathrm{th}}^{C}|=\sqrt{\left(1+c)/(1-c\right)}$, whereas if it exists at time *t* for the case 0<c≤1, the threshold value of the decoherence function satisfies $\left|F\left(t\right)|>\right|F_{\mathrm{th}}^{C}|=\sqrt{\left(1-c)/(1+c\right)}$. In both Markovian and non-Markovian dynamics regimes, there are no entanglement sudden death and rebirth phenomena for the case |c|=1, whereas for the case 0<|c|<1, the entanglement sudden death phenomenon occurs, and in the non-Markovian dynamics regime, the entanglement rebirth phenomenon can occur if the secondary maximum of the decoherence function is larger than the threshold value $|F_{\mathrm{th}}^{C}|$.

The time dependent maximum CHSH-Bell function B(t) for the initial states of the two-qubit system of Equation (22) can be reduced to

(24) $$B\left(t\right)=2\sqrt{{\left|F\left(t\right)\right|}^{4}+c^{2}}.$$

The presence of entanglement C(t)>0, namely, c≠0, is a necessary condition to achieve nonlocality. The initial CHSH-Bell function $B\left(0\right)=2\sqrt{1+c^{2}}>2$ corresponds to the fact that the two-qubit system always initially displays the quantum nonlocality. In a long time limit t→+∞, for the case |c|=1, B(+∞)=2, and thus the two-qubit system always displays quantum nonlocality. For the case 0<|c|<1 the threshold value of the decoherence function should satisfy $\left|F\left(t\right)|>\right|F_{\mathrm{th}}^{B}|=\sqrt[{4}]{1-c^{2}}$ to ensure that the CHSH-Bell function B(t) is larger than the classical threshold $B_{\mathrm{th}}$ and the nolocality of the two-qubit system undergoes the transition from quantum to classical.

The close relation between B(t) and C(t) for the two-qubit system prepared in the initial composite Bell states of Equation (22) can be expressed as

(25) $$B\left(t\right)=\left\{\begin{array}{cc} \frac{2}{1-c}\sqrt{{\left[2C(t)+1+c\right]}^{2}+c^{2}{\left(1-c\right)}^{2}}, & -1\leq c<0,\\ \frac{2}{1+c}\sqrt{{\left[2C(t)+1-c\right]}^{2}+c^{2}{\left(1+c\right)}^{2}}, & 0<c\leq 1. \end{array}\right.$$

For the case −1≤c<0, the classical threshold $C_{\mathrm{th}}$, which corresponds to the Bell function $B\left(t\right)>B_{\mathrm{th}}=2$ only exists for −1<c<0 and can be expressed as $C_{\mathrm{th}}=\left(1-c\right)\sqrt{1-c^{2}}/2-\left(1+c\right)/2$, whereas for c=−1, the maximum CHSH-Bell function B(t) is always larger than the threshold $B_{\mathrm{th}}=2$. Similarly, for the case 0<c≤1, the threshold $C_{\mathrm{th}}$ for the Bell function larger than the threshold $B_{\mathrm{th}}=2$ exists for 0<c<1 and can be expressed as $C_{\mathrm{th}}=\left(1+c\right)\sqrt{1-c^{2}}/2-\left(1-c\right)/2$, while the maximum CHSH-Bell function B(t) is always larger than the threshold $B_{\mathrm{th}}=2$ for c=1.

We now focus on the case that the two-qubit system is prepared for in a subclass of Bell-diagonal states, namely, the Werner states [1,107]

(26) $$\begin{array}{cc} \rho_{\psi }\left(0\right) & =r|\psi_{\pm }\left(0\right)\rangle \langle \psi_{\pm }\left(0\right)|+\frac{1-r}{4}I_{4},\\ \rho_{\varphi }\left(0\right) & =r|\varphi_{\pm }\left(0\right)\rangle \langle \varphi_{\pm }\left(0\right)|+\frac{1-r}{4}I_{4}, \end{array}$$

where 0≤r≤1 denotes the purity parameter of the initial states, and $I_{4}$ is the 4×4 identity matrix. The concurrence for the two-qubit system prepared in the Werner states initially of Equation (26) can be reduced to

(27) $$C^{\psi }\left(t\right)=C^{\varphi }\left(t\right)=\max\left\{{0,r|F\left(t\right)|}^{2}-\frac{1}{2}\left(1-r\right)\right\}.$$

The entanglement of the two-qubit system exists if the initial value of concurrence C(0) in the Werner states is larger than zero, correspondingly 1/3<r≤1. The concurrence exists at time *t* if the threshold value of the decoherence function satisfies $\left|F\left(t\right)|>\right|F_{\mathrm{th}}^{C}|=\sqrt{\left(1-r)/(2r\right)}$. The entanglement sudden death and rebirth phenomena only occur in non-Markovian dynamics regimes for the case r=1, whereas for the case 1/3<r<1, the entanglement sudden death phenomenon occurs in both Markovian and non-Markovian dynamics regimes. The entanglement rebirth phenomenon can occur if the secondary maximum of the decoherence function is larger than the threshold value $|F_{\mathrm{th}}^{C}|$ in the non-Markovian dynamics regime.

The time dependent maximum CHSH-Bell function B(t) for the initial Werner states of Equation (26) can be reduced to

(28) $$B\left(t\right)=2r\sqrt{{\left|F\left(t\right)\right|}^{4}+1}.$$

In the presence of entanglement C(t)>0, namely 1/3<r|≤1, if the initial CHSH-Bell function $B\left(0\right)=2\sqrt{2}r>2$, namely $\sqrt{2}/2<r\leq 1$, the two-qubit system displays quantum nonlocality initially. For the case r=1, B(+∞)=2 in long time limit t→+∞, and the two-qubit system always displays quantum nonlocality, whereas the two-qubit system exhibits the transition from quantum to classical nolocalities for the case $\sqrt{2}/2<r<1$, and the threshold value of the decoherence function satisfies $\left|F\left(t\right)|>\right|F_{\mathrm{th}}^{B}|=\sqrt[{4}]{1/r^{2}-1}$, provided that the CHSH-Bell function B(t) is larger than the classical threshold $B_{\mathrm{th}}$. The initial CHSH-Bell function B(0)≤2 and the two-qubit system always displays classical nonlocality for the case $1/3<r\leq \sqrt{2}/2$.

For the two-qubit system prepared initially in the Werner states of Equation (26), the close relation between B(t) and C(t) can be expressed as

(29) $$B\left(t\right)=2\sqrt{{\left[C\left(t\right)+\frac{1}{2}\left(1-r\right)\right]}^{2}+r^{2}}.$$

The classical threshold $C_{\mathrm{th}}$ corresponding to the Bell function $B\left(t\right)\geq B_{\mathrm{th}}=2$ can be expressed as $C_{\mathrm{th}}=\sqrt{1-r^{2}}-\left(1-r\right)/2$ which depends only on the purity parameter *r* of the initial states of Equation (26), and it is a decreasing function of the purity parameter *r*; for the presence of entanglement 1/3<r≤1, it satisfies $0\leq C_{\mathrm{th}}<\left(2\sqrt{2}-1\right)/3$.

## 3. Discussion

In this section, we demonstrate the numerical results of the non-Markovian disentanglement dynamics of a two-qubit system consisting of two noninteracting identical single qubits independently coupled to its local nonequilibrium environment. We mainly focus on how the environmental nonstationary and non-Markovian properties influence the non-Markovianity $N_{T}$, the entanglement quantified by the concurrence and the nonlocality characterized by the Bell function. The comparisons with the environmental stationary and memoryless cases are also discussed.

**Figure 1:**
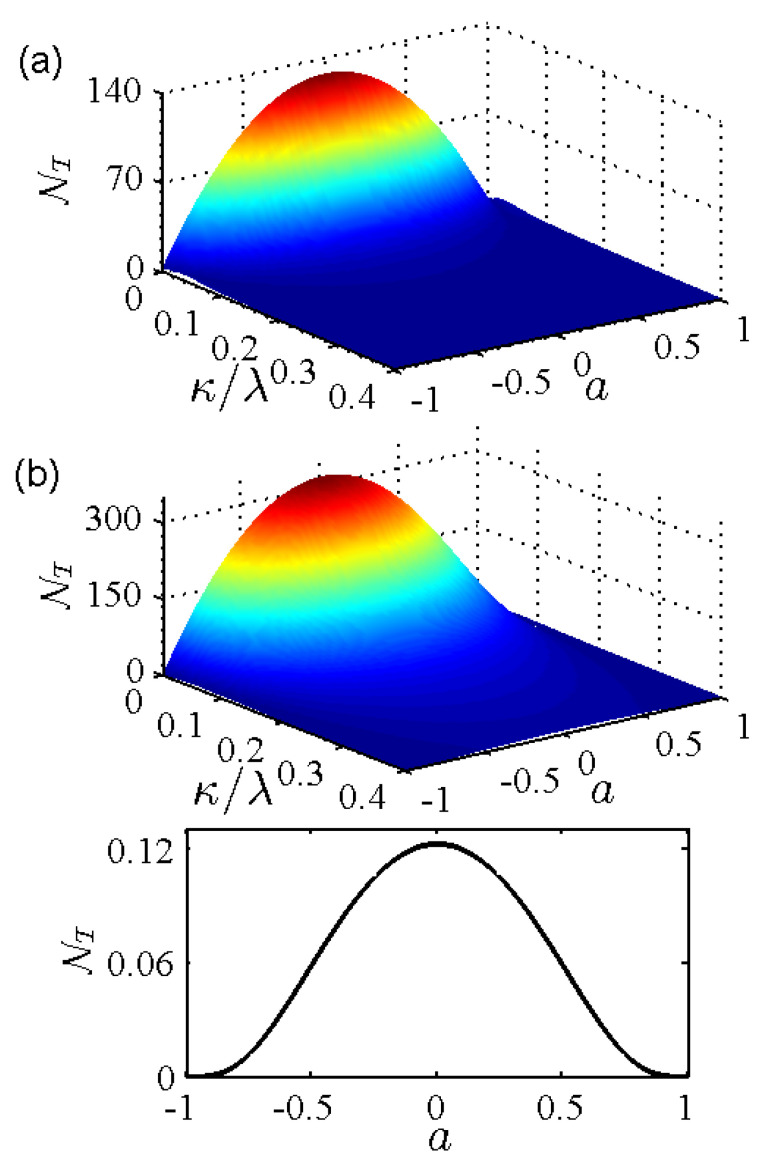
(Color online) Non-Markovianity $N_{T}$ of a two-qubit system in nonequilibrium environments as a function of the environmental memory decay rate κ and the nonstationary parameter *a* in (**a**) the weak coupling regime with ν/λ=0.8 and (**b**) the strong coupling regime with ν/λ=2. The bottom panel of (**b**) is for the memoryless case κ→+∞.

Figure 1 shows the non-Markovianity $N_{T}$ of a two-qubit system interacting with nonequilibrium environments as a function of the environmental memory decay rate κ and the nonstationary parameter *a*. Similar to the case of a single qubit system coupled to nonequilibrium environments, for a given value of the environmental memory decay rate κ, the non-Markovianity $N_{T}$ shows symmetrical behavior for positive and negative environmental nonstationary parameter *a* in both weak and strong coupling regimes. As the environmental nonstationary parameter *a* deviates from zero for a given environmental memory decay rate κ, the non-Markovianity $N_{S}$ decreases due to the suppression in the dynamical decoherence induced by the environmental nonequilibrium feature. In both weak and strong coupling regimes, for a given value of the environmental nonstationary parameter *a*, the non-Markovianity $N_{T}$ increases with the decrease in the environmental memory decay rate κ. The non-Markovianity $N_{T}$ decreases to zero as the environmental memory decay rate κ increases in the weak coupling regime, as shown in Figure 1a, whereas it does not decrease to zero in the strong coupling regime as displayed in Figure 1b.

**Figure 2:**
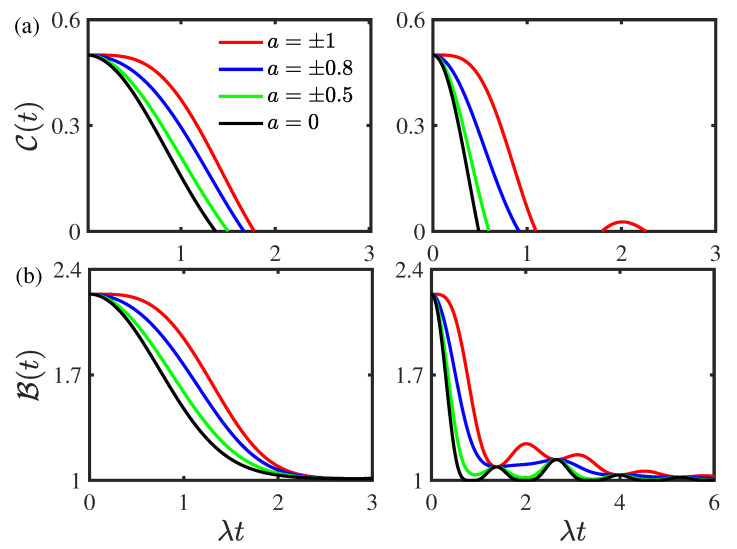
(Color online) Time evolution of (**a**) the concurrence C(t) and (**b**) the Bell function B(t) for different values of the environmental nonstationary parameter *a* for the two-qubit system prepared initially in the composite Bell states with the initial state parameter |c|=0.5. Left panel: the weak coupling regime with ν/λ=0.8. Right panel: the strong coupling regime with ν/λ=2. The environmental memory decay rate is given by κ/λ=1. The threshold value corresponding to the entanglement rebirth phenomenon in the strong coupling regime in the right panel of (**a**) is $|a_{\mathrm{th}}|=0.95$.

Figure 2 displays the time evolution of the concurrence C(t) and the Bell function B(t) for different values of the environmental nonstationary parameter *a* for the two-qubit system prepared initially in the composite Bell states. As shown in Figure 2a, the concurrence C(t) decays monotonically and there is an entanglement of the sudden death phenomenon in the weak coupling regime for both the nonstationary a≠0 and stationary a=0 cases. In the strong coupling regime, as the nonstationary parameter |a| increases, the concurrence C(t) undergoes a transition from monotonical decay to nonmonotonical decay with nonzero entanglement revivals. When the nonstationary parameter |a| is smaller than a certain threshold value $|a_{\mathrm{th}}|=0.95$, the entanglement only displays sudden death phenomenon and the rebirth phenomenon disappears. In both the weak and strong coupling regimes, the concurrence C(t) increases as the environmental nonstationary parameter *a* departs from zero. This indicates that the environmental nonequilibrium feature can suppress the disentanglement dynamics. As displayed in Figure 2b, in the weak coupling regime, the Bell function B(t) decays monotonically, whereas it shows nonmonotonical decays in the strong coupling regime. In both the weak and strong coupling regimes, the nolocality undergoes a transition from quantum to classical and it increases as the environmental nonstationary parameter *a* departs from zero. This reflects that the environmental nonequilibrium feature can enhance the quantum nonlocality. In addition, the environmental nonequilibrium feature does not influence the initial values of the concurrence C(0) and Bell function B(0) in both the weak and strong coupling regimes for the system prepared in the composite Bell states initially.

**Figure 3:**
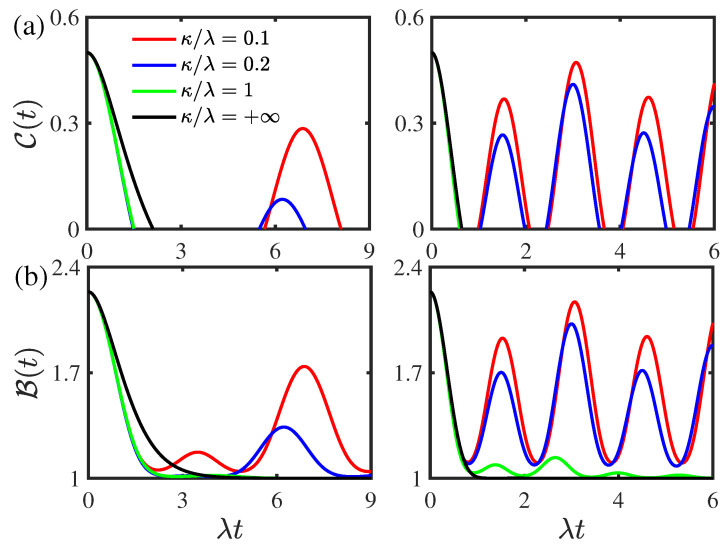
(Color online) Time evolution of (**a**) the concurrence C(t) and (**b**) the Bell function B(t) for different values of the environmental memory decay rate κ for the two-qubit system prepared initially in the composite Bell states with the initial state parameter |c|=0.5. Left panel: the weak coupling regime with ν/λ=0.8. Right panel: the strong coupling regime with ν/λ=2. The environmental nonstationary parameter is given by |a|=0.5. The threshold values corresponding to the entanglement rebirth phenomenon in the weak and strong coupling regimes in left and right panel of (**a**) are $\kappa_{\mathrm{th}}=0.27\lambda$ and $\kappa_{\mathrm{th}}=0.87\lambda$, respectively.

Figure 3 displays the time evolution of the concurrence C(t) and the Bell function B(t) for different values of the environmental memory decay rate κ for the two-qubit system prepared initially in the composite Bell states. As shown in Figure 3a, the concurrence C(t) undergoes a transition from nonmonotonical decay to monotonical decay as the environmental memory decay rate κ increases in both the weak and strong coupling regimes. The entanglement only displays sudden death phenomenon, and the rebirth phenomenon disappears when the environmental memory decay rate κ is larger than the threshold value $\kappa_{\mathrm{th}}=0.27\lambda$ and $\kappa_{\mathrm{th}}=0.87\lambda$ in the weak and strong coupling regimes, respectively. In the presence of entanglement rebirth phenomenon, the entanglement revivals in the concurrence C(t) become obvious as the environmental memory decay rate κ decreases in both the weak and strong coupling regimes. This indicates that the environmental non-Markovian feature can enhance the entanglement revivals and suppress the disentanglement dynamics. As displayed in Figure 3b, the Bell function B(t) undergoes a transition from nonmonotonical decay to monotonical decay in the weak coupling regime, whereas in the strong coupling regime, it decays nonmonotonically and it increases as the environmental memory decay rate κ decreases. This reflects that the environmental non-Markovian feature can enhance the quantum nonlocality in the strong coupling regime. In contrast, the decay of the Bell function B(t) exhibits a transition from nonmonotonical decay to monotonical decay with the increase in the environmental memory decay rate κ in the weak coupling regime.

**Figure 4:**
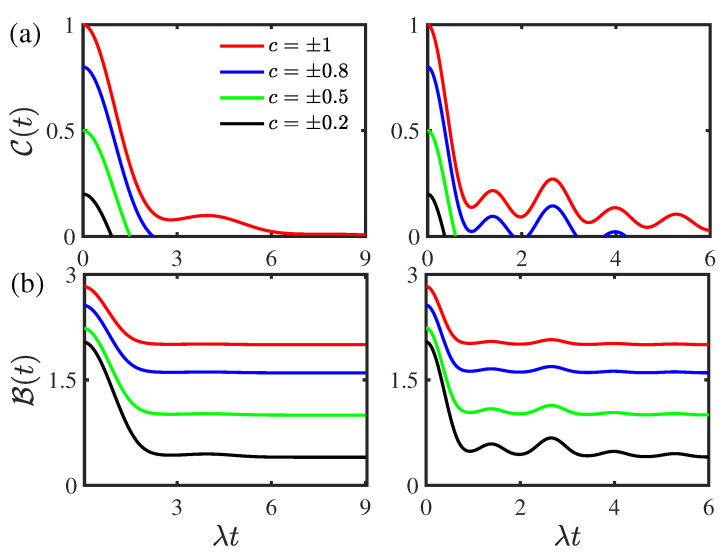
(Color online) Time evolution of (**a**) the concurrence C(t) and (**b**) the Bell function B(t) for the two-qubit system prepared initially in the composite Bell states for different values of the initial state parameter *c*. Left panel: the weak coupling regime with ν/λ=0.8. Right panel: the strong coupling regime with ν/λ=2. The environmental nonstationary parameter is given by |a|=0.5 and the environmental memory decay rate is given by κ/λ=1. The threshold value corresponding to the entanglement rebirth phenomenon in the strong coupling regime in right panel of (**a**) is $|c_{\mathrm{th}}|=0.57$.

Figure 4 displays the time evolution of the concurrence C(t) and the Bell function B(t) for different values of the initial state parameter *c* for the two-qubit system prepared initially in the composite Bell states. As shown in Figure 4a, the entanglement displays sudden death phenomenon in the weak couping regime, whereas it displays a transition from sudden death to rebirth for different initial state parameter |c| in the strong coupling regime; as the initial state parameter |c| is smaller than the threshold value $|c_{\mathrm{th}}|=0.57$, the entanglement only displays the sudden death phenomenon, and the rebirth phenomenon disappears. As the initial state parameter |c| increases, the concurrence C(t) increases in both the weak and strong coupling regimes, and the entanglement revivals in the concurrence C(t) become obvious in the strong coupling regime. This indicates that the initial state parameter can enhance quantum entanglement. As shown in Figure 4b, the nolocality undergoes a transition from quantum to classical as the initial state parameter |c| decreases from the threshold value $|c_{\mathrm{th}}|=1$ in both the weak and strong coupling regimes. Due to the non-Markovian behavior in the disentanglement dynamics, the Bell function B(t) decays nonmonotonically. In both the weak and strong coupling regimes, the Bell function B(t) increases as the initial state parameter |c| increases. This reflects that the initial state parameter can enhance nonlocality. Different from the fact that the environmental nonequilibrium feature does not influence the concurrence and Bell function initially, the initial values of the concurrence C(0) and Bell function B(0) depend closely on the initial state parameter |c| and they increase with the increase in the initial state parameter |c| in both the weak and strong coupling regimes. In both the weak and strong coupling regimes, the initial Bell function B(0) is always larger than the threshold $B_{\mathrm{th}}=2$ for an arbitrary initial state parameter |c| corresponding to the fact that the two-qubit system always displays quantum nonlocality initially for the two-qubit system prepared in the composite Bell states.

**Figure 5:**
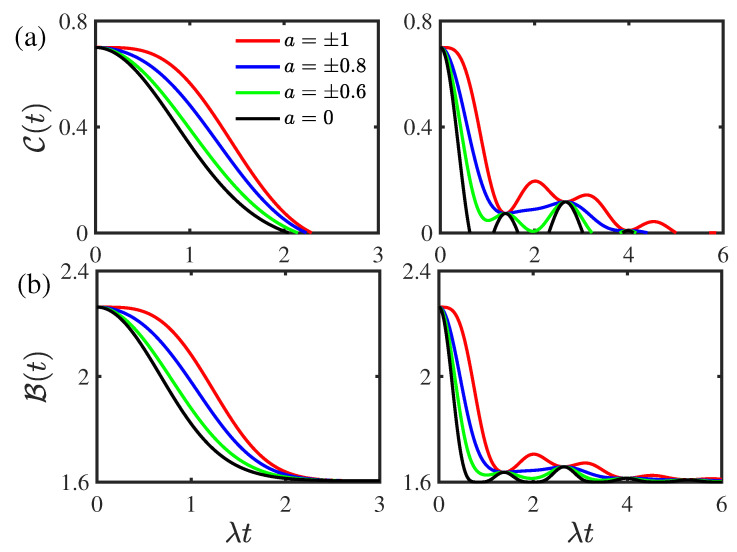
(Color online) Time evolution of (**a**) the concurrence C(t) and (**b**) the Bell function B(t) for different values of the environmental nonstationary parameter *a* for the two-qubit system prepared initially in the Werner states with the initial purity parameter r=0.8. Left panel: the weak coupling regime with ν/λ=0.8. Right panel: the strong coupling regime with ν/λ=2. The environmental memory decay rate is given by κ/λ=1.

Figure 5 shows the time evolution of the concurrence C(t) and the Bell function B(t) for different values of the environmental nonstationary parameter *a* for the two-qubit system prepared initially in the Werner states. Similar to the case that the two-qubit system initially prepared in the composite Bell states, as displayed in Figure 5a, the concurrence C(t) decays monotonically, and it exhibits entanglement sudden death phenomenon for both the nonstationary a≠0 and stationary a=0 cases in the weak coupling regime, whereas there are obvious entanglement sudden death and rebirth phenomena in the strong coupling regime. As shown in Figure 5b, the Bell function B(t) decays monotonically in the weak coupling regime, while it decays nonmonotonically in the strong coupling regime. It undergoes a transition between quantum and classical nonlocalities in both the weak and strong coupling regimes. As the environmental nonstationary parameter *a* derivates from zero, the concurrence C(t) and Bell function B(t) increase, whereas the initial values of the concurrence C(0) and Bell function B(0) do not change in both the weak and strong coupling regimes. This indicates that the environmental nonequilibrium feature can suppress the disentanglement dynamics and enhance the quantum nonlocality but it does not influence the initial concurrence C(0) and Bell function B(0). In addition, the influence of the environmental nonequilibrium feature on disentanglement dynamics and quantum nonlocality in the weak coupling regime is more obvious than that in the strong coupling regime.

**Figure 6:**
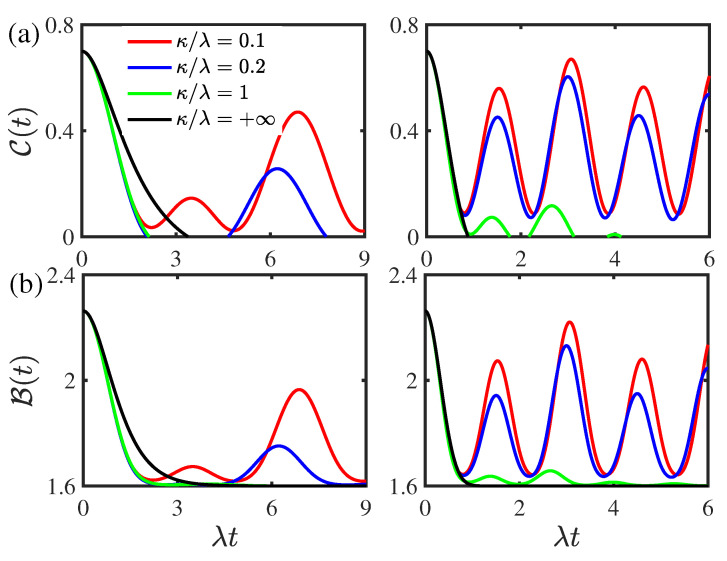
(Color online) Time evolution of (**a**) the concurrence C(t) and (**b**) the Bell function B(t) for different values of the environmental memory decay rate κ for the two-qubit system prepared initially in the extended Werner states with the initial purity parameter r=0.8. Left panel: the weak coupling regime with ν/λ=0.8. Right panel: the strong coupling regime with ν/λ=2. The environmental nonstationary parameter is given by |a|=0.5. The threshold values corresponding to the entanglement rebirth phenomenon in the weak and strong coupling regimes in left and right panel of (**a**) are $\kappa_{\mathrm{th}}=0.66\lambda$ and $\kappa_{\mathrm{th}}=1.50\lambda$, respectively.

Figure 6 displays the time evolution of the concurrence C(t) and the Bell function B(t) for different values of the environmental memory decay rate κ for the two-qubit system prepared initially in the extended Werner states. As displayed in Figure 6a, similar to the case that the two-qubit system initially prepared in the composite Bell states, the concurrence C(t) exhibits a transition from nonmonotonical decay to monotonical decay in both the weak and strong coupling regimes as the environmental memory decay rate κ increases. The entanglement only displays sudden death phenomenon and the rebirth phenomenon disappears when the environmental memory decay rate κ is larger than the threshold value $\kappa_{\mathrm{th}}=0.66\lambda$ and $\kappa_{\mathrm{th}}=1.50\lambda$ in the weak and strong coupling regimes, respectively. In the strong coupling regime, the entanglement revivals in the concurrence C(t) enhances as the environmental memory decay rate κ decreases. As shown in Figure 6b, the Bell function B(t) decays nonmonotonically, and for a given time *t* it decreases with the increase in the environmental memory decay rate κ in the strong coupling regime. In contrast, in the weak coupling regime, the Bell function B(t) exhibits a transition from nonmonotonical decay to monotonical decay as the environmental memory decay rate κ decreases and the Bell function B(t) decreases in some time intervals and increases in some other time intervals as the environmental memory decay rate κ decreases.

**Figure 7:**
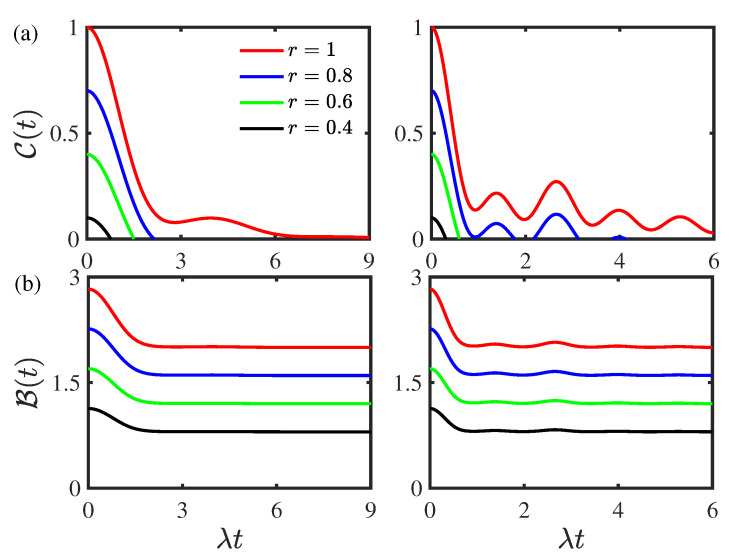
(Color online) Time evolution of (**a**) the concurrence C(t) and (**b**) the Bell function B(t) for the two-qubit system prepared initially in the Werner states for different values of the initial purity parameter *r*. Left panel: the weak coupling regime with ν/λ=0.8. Right panel: the strong coupling regime with ν/λ=2. The environmental nonstationary parameter is given by |a|=0.5 and the environmental memory decay rate is given by κ/λ=1. The threshold value corresponding to the entanglement rebirth phenomenon in the strong coupling regime in right panel of (a) is $r_{\mathrm{th}}=0.65$.

Figure 7 displays the time evolution of the concurrence C(t) and Bell function B(t) for different values of initial purity state parameter *r* for the two-qubit system prepared initially in the Werner states. As shown in Figure 7a, similar to the case that the two-qubit system initially prepared in the composite Bell states, as the initial purity state parameter *r* decreases from the threshold value $r_{\mathrm{th}}=1$, the entanglement sudden death phenomenon occurs in the weak coupling regime. In the strong coupling regime, the entanglement displays sudden death and rebirth phenomena, and it only shows sudden death phenomenon; the rebirth phenomenon disappears as the initial purity state parameter *r* is smaller than the threshold value $r_{\mathrm{th}}=0.65$. With the increase in the initial purity state parameter *r*, the concurrence C(t) increases in both the weak and strong coupling regimes and the entanglement revivals in the concurrence C(t) become obvious in the strong coupling regime. This reflects the fact that the initial purity state parameter *r* can enhance quantum entanglement. As shown in Figure 7b, the Bell function B(t) decays monotonically and nonmonotonically in the weak and strong coupling regimes, respectively. In both the weak and strong coupling regimes, the Bell function B(t) increases as the initial purity state parameter *r* increases. This indicates that the initial state parameter can enhance nonlocality. As the initial purity state parameter *r* decreases from the threshold value $r_{\mathrm{th}}=1$, it first undergoes a transition from quantum nonlocality to classical nonlocality and then it only displays classical nonlocality when the initial purity state parameter *r* is smaller than the threshold value $r_{\mathrm{th}}=\sqrt{2}/2$ due to the fact that the initial Bell function B(0) is not always larger than the threshold $B_{\mathrm{th}}=2$ for the two-qubit system prepared initially in the Werner states. This is quite different from the case that the two-qubit system prepared initially in the composite Bell states.

**Figure 8:**
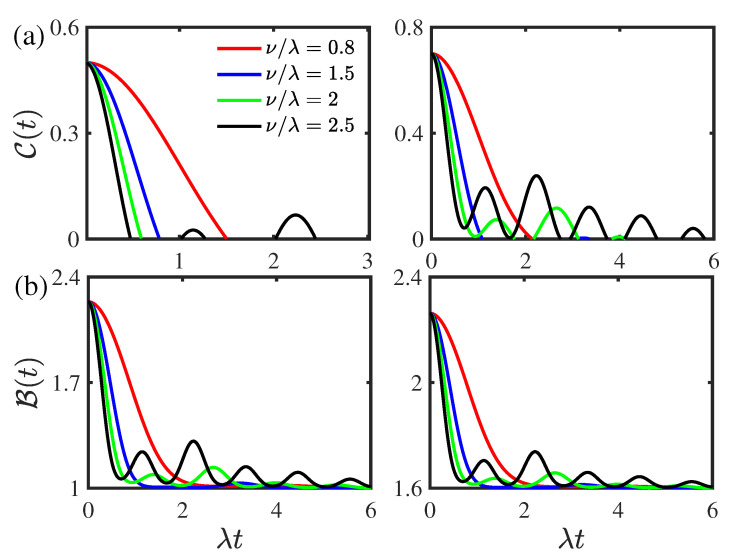
(Color online) Time evolution of (**a**) the concurrence C(t) and (**b**) the Bell function B(t) different values of the coupling strength ν. Left panel: for the two-qubit system prepared initially in the composite Bell states with the initial state parameter |c|=0.5. Right panel: for the two-qubit system prepared initially in the Werner states with the initial purity parameter r=0.8. The environmental nonstationary parameter is given by |a|=0.5 and the environmental memory decay rate is given by κ/λ=1. The threshold values corresponding to the entanglement rebirth phenomenon in the composite Bell states and in the Werner states in left and right panel of (**a**) are $\nu_{\mathrm{th}}=2.2\lambda$ and $\nu_{\mathrm{th}}=1.47\lambda$, respectively.

Figure 8 shows the time evolution of the concurrence C(t) and Bell function B(t) for different values of the coupling strength ν for the two-qubit system prepared initially in the composite Bell states and Werner states, respectively. As displayed in Figure 8a, for the weak coupling case (small ν), the entanglement shows sudden death phenomenon; as the coupling strength ν increases, the entanglement rebirth phenomenon occurs for the two-qubit system initially prepared in the composite Bell states and Werner states. The threshold values corresponding to the entanglement rebirth phenomenon in the composite Bell states and in the Werner states are $\nu_{\mathrm{th}}=2.2\lambda$ and $\nu_{\mathrm{th}}=1.47\lambda$, respectively. In addition, as the coupling strength ν increases, the entanglement revivals in the concurrence C(t) become more obvious. This indicates that the coupling strength can enhance quantum entanglement. As shown in Figure 8b, the Bell function B(t) undergoes a transition from quantum nonlocality to classical nonlocality for the two-qubit system initially prepared in both the composite Bell states and Werner states. Furthermore, the Bell function B(t) decays monotonically and nonmonotonically for small and large values of the coupling strength ν, respectively. The Bell function B(t) decreases as the coupling strength ν increases. This reflects that the coupling strength can suppress nonlocality.

## 4. Conclusions

We have theoretically studied the disentanglement dynamics of a two-qubit system in the presence of nonequilibrium environments with nonstationary and non-Markovian RTN statistical properties. The reduced density matrix of the two-qubit system can be expressed in terms of the Kraus representation by means of the tensor products of the single qubit Kraus operators. We have derived the relations between the decoherence function and the entanglement characterized by the concurrence and the nonlocality quantified by the Bell function of the two-qubit system. We have identified the threshold values of the decoherence function to ensure the existences of the concurrence and nonlocal quantum correlations for a given evolution time when the two-qubit system is initially prepared in the composite Bell states and the Werner states, respectively. The results demonstrate that the environmental nonequilibrium feature can suppress the disentanglement of the two-qubit system and reduce the entanglement revivals in the two-qubit disentanglement dynamics. In addition, the environmental nonequilibrium feature can enhance the nonlocality in the two-qubit system. Moreover, the phenomena of entanglement sudden death and rebirth and the transition between quantum and classical nonlocalities closely depend on the parameters of the initial states and the environmental parameters, such as the nonstationary parameter, the memory decay rate and the coupling strength of the environmental noise. Our results are helpful for further understanding the quantum dynamics in nonequilibrium environments.

## Data Availability

Not applicable.

## Abbreviations

The following abbreviations are used in this manuscript:

RTN	Random telegraph noise
CHSH	Clauser-Horne-Shimony-Holt
CK	Chapman-Kolmogorov

## Appendix A. Generalized RTN Process Based on Classical Probability Theory

Based on the classical probability theory [95], a stochastic process ξ(t) is completely determined by an infinity hierarchy of the multi-point probability distribution

(A1) $$\begin{array}{cc} P_{n} & =P\left(\xi_{1},t_{1};\cdots ;\xi_{n},t_{n}\right)=\langle \delta (\xi_{1}-\xi \left(t_{1}\right))\cdots \delta (\xi_{n}-\xi \left(t_{n}\right))\rangle , \end{array}$$

which represents that the stochastic process ξ(t) takes the valve $\xi_{1}$ at time $t_{1}$, ⋯, and the value $\xi_{n}$ at time $t_{n}$ for all ordered sets of time $t_{1}>\cdots >t_{n}$(n≥1). The *n*-point joint probability $P_{n}$ obeys the following four Kolmogorov consistency conditions:

(1) Nonnegative— i.e., $P_{n}\geq 0$;
(2) Normalization—i.e., $\sum_{\xi_{1}}P_{1}=1$;
(3) Symmetry—i.e., $P_{n}$ does not change by interchanging arbitrary pairs $\left(\xi_{k},t_{k}\right)$ and $\left(\xi_{l},t_{l}\right)$;
(4) Relation between $P_{n}$ and $P_{n-1}$—i.e., $\sum_{\xi_{n}}P_{n}=P_{n-1}$.

In general, the initial one-point probability distribution $P(\xi_{0},0)$ is given and if we want to obtain $P_{n}$, we should also know the conditional probability

(A2) $$\begin{array}{cc} P_{1|n-1} & =P\left(\xi_{1},t_{1}|\xi_{2},t_{2};\cdots ;\xi_{n},t_{n}\right)={\langle \delta (\xi_{1}-\xi \left(t_{1}\right))\rangle }_{\xi \left(t_{2}\right)=\xi_{2},\cdots ,\xi \left(t_{n}\right)=\xi_{n}}, \end{array}$$

which is the probability that the stochastic process ξ(t) at time $t_{1}$ has the valve $\xi_{1}$ under the condition that the stochastic process ξ(t) takes the valve $\xi_{2}$ at time $t_{2}$, ⋯, and the value $\xi_{n}$ at time $t_{n}$. The conditions of nonnegativity and normalization are satisfied

(A3) $$\begin{array}{c} P_{1|n-1}\geq 0,\sum_{\xi_{1}}P_{1|1}=1. \end{array}$$

A stochastic process ξ(t) is considered to be stationary if all $P_{n}$ depend only on the time difference

(A4) $$P\left(\xi_{1},t_{1}+\tau ;\cdots ;\xi_{n},t_{n}+\tau \right)=P\left(\xi_{1},t_{1};\cdots ;\xi_{n},t_{n}\right).$$

A necessary but not sufficient condition is that $P_{1}$ is independent of time. Equivalently, if there is at least one joint probability, $P_{i}$ satisfies

(A5) $$P\left(\xi_{1},t_{1}+\tau ;\cdots ;\xi_{i},t_{i}+\tau \right)\neq P\left(\xi_{1},t_{1};\cdots ;\xi_{i},t_{i}\right),$$

the stochastic process ξ(t) is nonstationary. A sufficient but not necessary condition is that $P_{1}$ is time-dependent.

A stochastic process ξ(t) is regarded to be Markovian if all $P_{1|n-1}$ satisfy

(A6) $$\begin{array}{c} P\left(\xi_{1},t_{1}|\xi_{2},t_{2};\cdots ;\xi_{n},t_{n}\right)=P\left(\xi_{1},t_{1}|\xi_{2},t_{2}\right). \end{array}$$

That is the probability for the stochastic process ξ(t) at time $t_{1}$ to take the valve $\xi_{1}$ under the condition that the stochastic process ξ(t) has the valve $\xi_{2}$ at time $t_{2}$, ⋯, and the value $\xi_{n}$ at time $t_{n}$ depends only on the last previous value $\xi_{2}$ at time $t_{2}$. $P_{1|1}$ is also called the conditional transition probability. It is remarkable that for a Markovian process, we can reconstruct an arbitrary multi-point probability distribution by means of the initial one-point distribution $P(\xi_{0},0)$ and conditional transition probability $P_{1|1}$ as

(A7) $$P\left(\xi_{1},t_{1};\cdots ;\xi_{n},t_{n}\right)=\prod_{i}^{n-1}P\left(\xi_{i},t_{i}|\xi_{i+1},t_{i+1}\right)P\left(\xi_{n},t_{n}\right),$$

where the one-point probability distribution satisfies

(A8) $$P\left(\xi_{1},t_{1}\right)=P\left(\xi_{1},t_{1}|\xi_{0},0\right)P\left(\xi_{0},0\right).$$

A necessary but not sufficient condition for a Markov process is that the conditional transition probability obeys the Chapman-Kolmogorov (CK) equation

(A9) $$P\left(\xi_{1},t_{1}|\xi_{3},t_{3}\right)=\sum_{\xi_{2}}P\left(\xi_{1},t_{1}|\xi_{2},t_{2}\right)P\left(\xi_{2},t_{2}|\xi_{3},t_{3}\right).$$

A stochastic process ξ(t) is non-Markovian if there is at least one conditional probability. $P_{1|i-1}$ depends not only on the last previous value $\xi_{i-1}$ at time $t_{i-1}$ but on one or more previous values $\xi_{j}$ at earlier time $t_{j}$ (j<i−1). A sufficient but not necessary condition is that the CK equation (A9) fails.

The subensemble of non-Markovian and nonstationary homogeneous stochastic processes can be extracted from subensembles of Markovian and stationary stochastic processes [95]. A simple assumption is that $P_{1}$ is time-dependent

(A10) $$P\left(\xi ,t\right)=\int P\left(\xi ,t|\xi_{0},0\right)P\left(\xi_{0},0\right)d\xi_{0},$$

with the initial nonstationary distribution $P(\xi_{0},0)$ and the conditional probability $P_{1|1}$ depends on its previous history

(A11) $$\frac{\partial }{\partial t}P\left(\xi ,t|\xi^{\prime },t^{\prime }\right)=\int_{t^{\prime }}^{t}K\left(t-\tau \right)M_{\xi }P\left(\xi ,\tau |\xi^{\prime },t^{\prime }\right)d\tau ,$$

where the initial condition is given by $P\left(\xi ,t^{\prime }|\xi^{\prime },t^{\prime }\right)=\delta \left(\xi -\xi^{\prime }\right)$, K(t−τ) denotes the memory kernel composite environmental noise ξ(t) and $M_{\xi }$ is a differential operator only involving derivatives with respect to ξ. Physically, the extraction of a subensemble nonstationary and non-Markovian stochastic processes with memory effect and initial nonstationary distribution taken into account means that the environment is in a certain nonequilibrum state initially and the statistical properties of the environmental noise depend on previous history. For the case a=0, the environmental noise only displays stationary property and the environment is in equilibrium [87,88]. When the environmental noise is memoryless, i.e., K(t−τ)=δ(t−τ), the non-Markovian RTN recovers the Markovian one and its memory effect vanishes.

## Appendix B. Entanglement and Quantum Nonlocality of a Two-Qubit System

In this appendix, we introduce the most commonly used measures of the entanglement and quantum nonlocality of a two-qubit system.

For a two-qubit system, all the entanglement measures are compatible, and we can use the concurrence to quantify the entanglement defined as [94,102]

(A12) $$C\left(t\right)=\max\left\{0,\sqrt{\lambda_{1}\left(t\right)}-\sqrt{\lambda_{2}\left(t\right)}-\sqrt{\lambda_{3}\left(t\right)}-\sqrt{\lambda_{4}\left(t\right)}\right\},$$

where $\lambda_{i}\left(t\right)$ are the eigenvalues of the matrix $\zeta \left(t\right)=\rho \left(t\right)\left(\sigma_{y}⊗\sigma_{y}\right)\rho^{*}\left(t\right)\left(\sigma_{y}⊗\sigma_{y}\right)$ arranged in decreasing order with $\rho^{*}\left(t\right)$ denoting the complex conjugation of the two-qubit reduced density matrix ρ(t) in the two-qubit basis $B_{T}$. The concurrence C(t) varies from the maximum 1 for a maximally entangled state to the minimum 0 for a completely disentangled state.

For pure quantum state, the entanglement corresponds to nonlocal correlations, whereas it is not the general case for mixed states due to the fact that the environmental noise gives rise to the decay of nonlocal correlations. The nonlocality can be identified by the violation of the Bell inequalities in the presence of entanglement (C(t)>0). The Clauser-Horne-Shimony-Holt (CHSH) form of Bell function has been widely used to determine whether there are nonlocal correlations of the entangled system. The maximum Bell function B(t) for an entangled two-qubit system can be, based on the Horodecki criterion, expressed as [103]

(A13) $$B\left(t\right)=2\sqrt{\max_{j>k}\left[\mu_{j}\left(t\right)+\mu_{k}\left(t\right)\right]},$$

where the subscripts j,k=1,2,3 and $\mu_{j}\left(t\right)$ and $\mu_{k}\left(t\right)$ are functions in terms of the elements of the two-qubit reduced density matrix. If the Bell function B(t) is larger than the classical threshold $B_{\mathrm{th}}=2$, the quantum correlations of the entangled two-qubit system cannot be reproduced by any classical local model.

It is known that the Bell states and Werner mixed states of a two-qubit system play an essential role in quantum computation and quantum information [6]. The two-qubit reduced density matrix expressed in Equation (17) for initial composite Bell states and Werner states has an *X* structure both initially and during the dynamical evolution. The concurrence C(t) for an initial *X* structure reduced density matrix of a two-qubit system can be computed in a particular form as [104]

(A14) $$C_{X}\left(t\right)=\max\left\{0,C_{1}\left(t\right),C_{2}\left(t\right)\right\},$$

where

(A15) $$\begin{array}{cc} C_{1}\left(t\right) & =2\left[|\rho_{23}\left(t\right)|-\sqrt{\rho_{11}\left(t\right)\rho_{44}\left(t\right)}\right],\\ C_{2}\left(t\right) & =2\left[|\rho_{14}\left(t\right)|-\sqrt{\rho_{22}\left(t\right)\rho_{33}\left(t\right)}\right]. \end{array}$$

The time dependent maximum CHSH-Bell function B(t) for an *X* structure two-qubit density matrix can be expressed analytically as [105]

(A16) $$B_{X}\left(t\right)=\max\left\{B_{1}\left(t\right),B_{2}\left(t\right)\right\},$$

where $B_{1}\left(t\right)=2\sqrt{\mu_{1}\left(t\right)+\mu_{2}\left(t\right)}$ and $B_{2}\left(t\right)=2\sqrt{\mu_{1}\left(t\right)+\mu_{3}\left(t\right)}$ with

(A17) $$\begin{array}{cc} \mu_{1}\left(t\right) & =4{\left[|\rho_{14}\left(t\right)|+|\rho_{23}\left(t\right)|\right]}^{2},\\ \mu_{2}\left(t\right) & ={\left[\rho_{11}\left(t\right)+\rho_{44}\left(t\right)-\rho_{22}\left(t\right)-\rho_{33}\left(t\right)\right]}^{2},\\ \mu_{3}\left(t\right) & =4{\left[|\rho_{14}\left(t\right)|-|\rho_{23}\left(t\right)|\right]}^{2}. \end{array}$$
